# Mechanisms for the retention of inorganic N in acidic forest soils of southern China

**DOI:** 10.1038/srep02342

**Published:** 2013-08-02

**Authors:** Jin-bo Zhang, Zu-cong Cai, Tong-bin Zhu, Wen-yan Yang, Christoph Müller

**Affiliations:** 1 School of Geography Sciences, Nanjing Normal University, Nanjing 210047, China; 2 State Key Laboratory of Soil and Sustainable Agriculture, Institute of Soil Science, Chinese Academy of Sciences; 3 Department of Plant Ecology, Justus-Liebig University Giessen, Heinrich-Buff-Ring 26, 35392 Giessen, Germany

## Abstract

The mechanisms underlying the retention of inorganic N in acidic forest soils in southern China are not well understood. Here, we simultaneously quantified the gross N transformation rates of various subtropical acidic forest soils located in southern China (southern soil) and those of temperate forest soils located in northern China (northern soil). We found that acidic southern soils had significantly higher gross rates of N mineralization and significantly higher turnover rates but a much greater capacity for retaining inorganic N than northern soils. The rates of autotrophic nitrification and NH_3_ volatilization in acidic southern soils were significantly lower due to low soil pH. Meanwhile, the relatively higher rates of NO_3_^−^ immobilization into organic N in southern soils can counteract the effects of leaching, runoff, and denitrification. Taken together, these processes are responsible for the N enrichment of the humid subtropical forest soils in southern China.

Most temperate forest ecosystems appear to be N-limited, while many humid subtropical and tropical forests may be naturally N-enriched^1^,^2^,^3^,^4^,^5^,^6^. The N status of relatively young temperate soils may differ markedly from that of highly weathered subtropical-tropical ecosystems^3^,^4^. Humid subtropical and tropical forest soils are generally characterized by rapid N cycling rates and high N availability^7^. Thus, the capacity of a soil to conserve inorganic N is critical for humid subtropical and tropical soils to achieve N enrichment. The quantities of the different forms of inorganic N in soils are fundamentally controlled by the gross N transformation rates^8^. Previous investigations have demonstrated that humid subtropical and tropical acidic soils have low autotrophic and relatively high heterotrophic nitrification rates^9^,^10^. It has been shown that NO_3_^−^ could be retained via dissimilatory NO_3_^−^ reduction to ammonia (DNRA) in forest soils in a region in Chile with very high rainfall^11^. High mineral N production can be combined with efficient N immobilization in soils^12^. Humid subtropical forest soils also have weaker denitrification abilities than humid temperate forest soils^13^. A mechanistic understanding of N dynamics in various ecosystems requires identifying and quantifying the N-pool-specific and process-specific gross N transformation rates (e.g., immobilization, DNRA, autotrophic nitrification, and organic N oxidation processes)^14^,^15^. To date, the mechanisms underlying the retention of inorganic N in humid subtropical forest soils have not been quantitatively investigated in detail based on N transformation dynamics.

## Results

The soil pH ranged from 4.1 to 7.4 (Table 1). Two samples collected from northern China (soils 7, 8) had pH values > 6.0, three samples collected from northern China (soils 1, 4, and 6) had pH values between 5.0 and 6.0, and the remaining samples had pH values < 5.0. The pH in northern soils (5.6 ± 1.0) was significantly higher than that in southern soils (4.4 ± 0.2; p < 0.05). Soil organic C (SOC) ranged from 21.4 to 83.0 g kg^−1^ for northern soils and from 13.2 to 68.2 g kg^−1^ for southern soils. The inorganic N was dominated by NH_4_^+^ in southern soils (the average ratio of NH_4_^+^/NO_3_^−^ was 8.2 for laboratory incubation and 3.9 for field incubation), while the NH_4_^+^ contents were similar to the NO_3_^−^ contents of northern soils (the average ratio of NH_4_^+^/NO_3_^−^ was 0.9).

We used a combination of ^15^N tracing experiments and full process-based N cycle models to quantify process-specific and pool-specific N transformation rates. Our results showed that compared to northern soils, the humid subtropical acidic forest soils of southern China had significantly higher gross rates of N mineralization (3.04 ± 1.03 mg N kg^−1^ d^−1^ vs 1.80 ± 0.50 mg N kg^−1^ d^−1^; p < 0.05) and a significantly higher turnover rate (554 ± 307 d vs 2519 ± 1419 d; p < 0.01). However, southern soils had a much higher capacity for retaining inorganic N than northern soils, as indicated by their significantly lower (p < 0.05) autotrophic nitrification rates (0.14 ± 0.17 mg N kg^−1^ d^−1^ vs 1.07 ± 1.57 mg N kg^−1^ d^−1^) and significantly higher (p < 0.01) rates of NO_3_^−^ immobilization into organic N (0.65 ± 0.41 mg N kg^−1^ d^−1^ vs 0.04 ± 0.11 mg N kg^−1^ d^−1^), which afforded them protection against N loss from leaching, runoff, and denitrification. We proposed that the mechanisms for retaining inorganic N in acidic forest soils in southern China is a combination of higher N production and much stronger capacities for the immobilization of inorganic N.

### Gross N transformation

**Figure 1:**
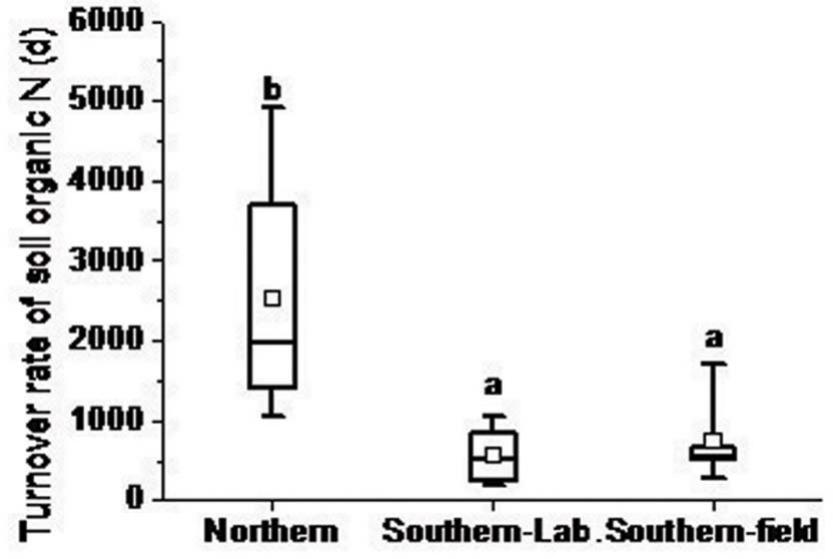
Box-whisker plot of turnover rates for organic N in northern and southern forest soils. In each box, there are five horizontal lines. From bottom to top, the lines represent the minimum, the middle number for the first half of the data set, the middle number for the whole list, the middle number for the second half of the data set, and the largest value, respectively. The square in each box is the average turnover rate. Identical letters indicate no significant difference. Southern-Lab denotes gross N transformation rates that were determined by laboratory incubation; Southern-field denotes gross N transformation rates that were determined by field incubation.

The gross N mineralization rates, as determined by laboratory incubation, varied from 1.52 to 4.63 mg N kg^−1^ d^−1^ in southern soils and were significantly lower in northern soils (from 1.02 to 2.29 mg N kg^−1^ d^−1^). No significant difference was observed between the gross mineralization rates obtained from the laboratory and those from the field experiments in southern soils. There was no significant relationship between gross mineralization rates and any individually measured soil property (Table 1, i.e., soil organic C, total N, pH, and C/N ratio). However, the turnover rate of organic N, which was obtained by calculating total N divided by the gross mineralization rate, was significantly slower in northern soils (2,519 ± 1,419 d) than in southern soils (554 ± 307 d). Turnover rates in southern soils, as determined by laboratory incubation, were comparable to results obtained using field-incubated soils (740 ± 554 d; Fig. 1). NH_4_^+^ immobilization ranged from 0 to 3.74 mg N kg^−1^ d^−1^ (with an average of 1.8 mg N kg^−1^ d^−1^) in southern soils and from 0 to 2.58 mg N kg^−1^ d^−1^ (with an average of 0.59 mg N kg^−1^ d^−1^) in northern soils, but the difference was only marginally significant (p = 0.053). The immobilization rates of NH_4_^+^ were positively correlated with gross mineralization rates (R^2^ = 0.55, p < 0.001).

The gross autotrophic nitrification rates, as determined by laboratory incubation, ranged from 0 to 0.50 mg N kg^−1^ d^−1^ (with an average of 0.14 mg N kg^−1^ d^−1^) in southern acidic forest soils (Table 2), which were significantly lower than the rates obtained for northern soils (0.07–4.53 mg N kg^−1^ d^−1^, with an average of 1.07 mg N kg^−1^ d^−1^; p < 0.05). There was no significant difference in the gross autotrophic nitrification rates determined in the laboratory or in the field incubation in southern soils. There was a significant exponential relationship between soil pH and the gross autotrophic nitrification rate (R^2^ = 0.39; p < 0.01). The nitrification capacity (NC, the ratio of autotrophic nitrification to mineralization) in southern soils (0.05 ± 0.05 for laboratory incubation and 0.08 ± 0.04 for field incubation) was substantially lower than that found in northern soils (0.68 ± 0.96; p < 0.05). The NC was also significantly correlated with soil pH (R^2^ = 0.44; p < 0.01). The average heterotrophic nitrification rate in northern soils (0.25 ± 0.30 mg N kg^−1^ d^−1^) was lower than that in the southern samples that were incubated in the laboratory (0.70 ± 0.45 mg N kg^−1^ d^−1^), which was comparable to results obtained from field-incubated soil (0.48 ± 0.43 mg N kg^−1^ d^−1^).

DNRA rates were low (with an average of 0.08 mg N kg^−1^ d^−1^ for all soils), and the rates were not significantly different in the southern and northern forest soils tested by laboratory incubation. However, the DNRA rates measured after field incubation were substantially higher than those measured after laboratory incubation. Relatively high DNRA rates were observed in soils 17 and 18 (Table 2). The NO_3_^−^ immobilization rates were significantly different for southern and northern forest soils (p < 0.05). Northern soils, except soil 6, which exhibited a NO_3_^−^ immobilization rate of 0.31 mg N kg^−1^ d^−1^, had no ability to immobilize NO_3_^−^ (Table 2). In contrast, all 18 southern soils were able to immobilize NO_3_^−^, with values ranging from 0.11 mg N kg^−1^ d^−1^ to 1.71 mg N kg^−1^ d^−1^ (with an average of 0.60 mg N kg^−1^ d^−1^), irrespective of whether these rates were determined during laboratory or field incubation. Similar to the NO_3_^−^ immobilization rates, the total mineral N immobilization rates (NH_4_^+^ immobilization + NO_3_^−^ immobilization) in southern soils (from 0.59 to 6.42 mg N kg^−1^ d^−1^, with an average of 2.45 mg N kg^−1^ d^−1^) were also higher than those in northern soils (from 0 to 2.58 mg N kg^−1^ d^−1^, with an average of 0.63 mg N kg^−1^ d^−1^; p < 0.05).

**Figure 2:**
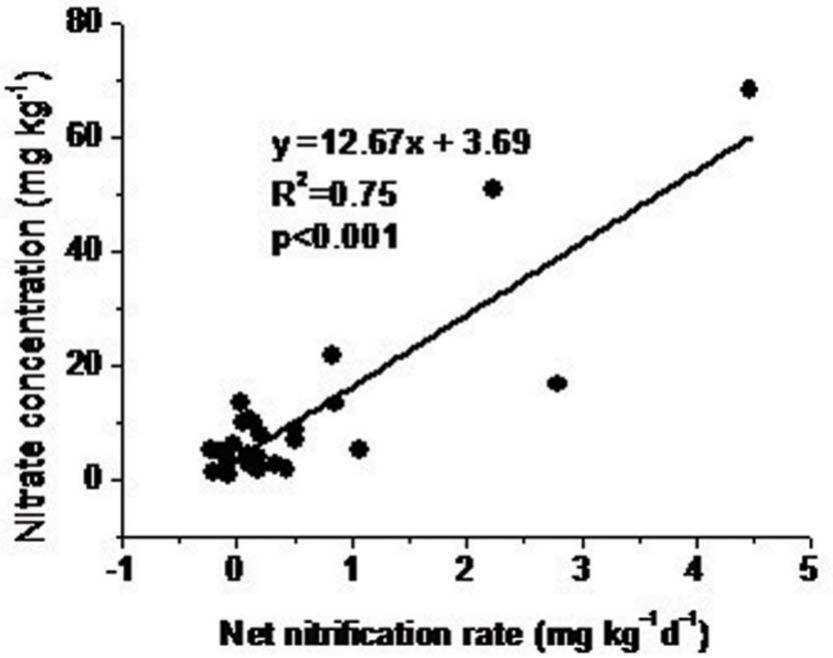
The relationship between nitrate concentration and net nitrification rate. Net nitrification rate was calculated by adding the nitrate production rates (autotrophic and heterotrophic nitrification) and subtracting the total nitrate consumption rates (immobilization and DNRA).

The NO_3_^−^ retention ability (NR), i.e., the ratio of NO_3_^−^ consumption (NO_3_^−^ immobilization [I_NO3_] and DNRA) to total nitrification rate, ranged from 0 to 0.57 (with an average of 0.24) in northern soils and from 0.56 to 2.17 (with an average of 1.27 for laboratory-incubated soils and 0.80 for field-incubated soils) in southern soils. The differences were significant (p < 0.001), while the differences between the NR of southern soils incubated in the laboratory vs field were not (Table 2). The net NO_3_^−^ production rate, which was calculated by taking the total NO_3_^−^ production rate (autotrophic nitrification + heterotrophic nitrification) minus the total NO_3_^−^ consumption rate (NO_3_^−^ immobilization + DNRA), was very useful for predicting NO_3_^−^ concentrations (Fig. 2).

## Discussion

In this investigation, we used a combination of ^15^N tracing experiments and full process-based N cycle models to quantify process-specific and pool-specific N transformation rates. Samples 14 and 15 were from the same core, but the ^15^N tracing experiment was carried out in the laboratory for soil 14 (added 50 μg N g^−1^ soil) and in the field for soil 15 (added 2 μg N g^−1^ soil). The N transformation rates measured in the laboratory and field were comparable (Table 2), indicating that N transformations were not stimulated by applying more tracer under laboratory conditions in warm, humid subtropical acidic forest soils from southern China.

**Figure 3:**
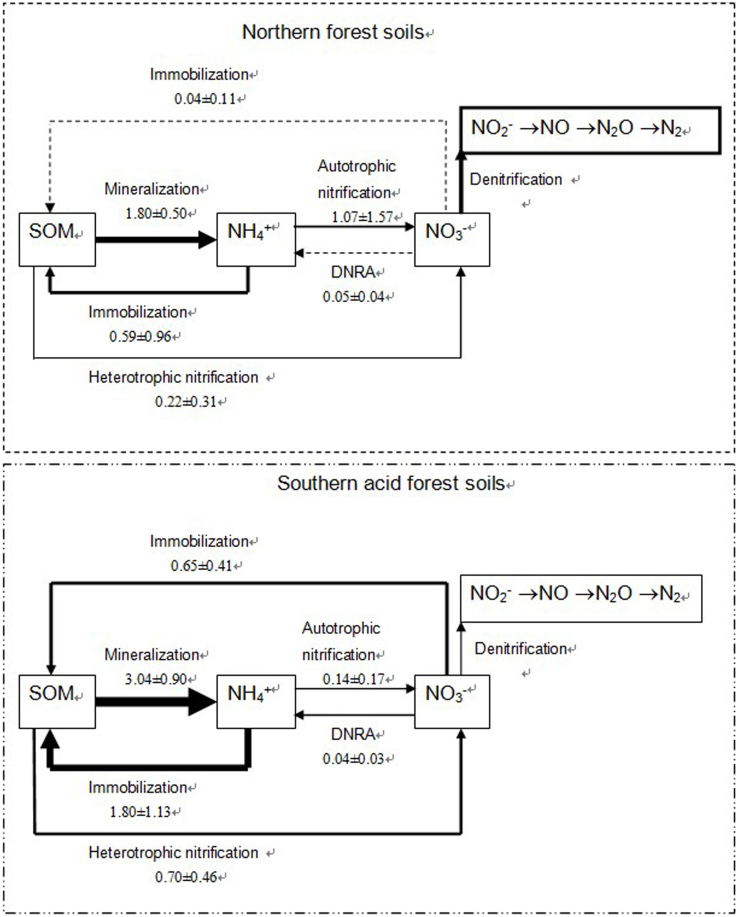
Conceptual model for the nitrogen cycle in northern and southern forest soils. The thickness of the arrows represents the relative importance of each flux. The data in the figure are the mean gross rates of N transformation, as determined by laboratory incubation, ±SD (mg N kg^−1^ d^−1^).

Our results underscored the fact that N transformation characteristics are responsible for the retention of inorganic N and result in the natural N enrichment of southern China's subtropical forest soils (hereafter called southern soil) (Fig. 3). Mineralization rates are higher in southern soils than in the temperate forest soils located in northern China (hereafter called northern soil), resulting in high N availability. Meanwhile, inorganic N is retained effectively in southern soils. The low soil pH suppresses ammonium oxidation and volatilization; thus, the inorganic N is dominated by ammonium. Southern soils also exhibit significant NO_3_^−^ retention capacity via conversion into organic N (immobilization) and NH_4_^+^ (DNRA) pools. Thus, NO_3_^−^ produced from nitrification can be conserved efficiently, counteracting denitrification, leaching or runoff. All of these processes serve to enrich southern forest soils in N.

In this study, the comparison of southern and northern soils is confined to particular regions, one data set, and partly confounded by soil pH, the quantity and quality of soil organic C and potentially by other unknown factors. Previous studies have suggested that most of the available N is immobilized by soil organic matter, which is the largest N pool in soil. Thus, the decomposition of soil organic matter primarily determines N mineralization and primary productivity^16^. Previous studies have also shown that N availability is high in humid subtropical and tropical forest soils due to the rapid rate of N cycling^1^,^2^,^3^,^4^,^5^,^6^. Mineralization rates in the majority of the subtropical soils (southern soils) examined in this study were slightly lower than those previously observed in some subtropical-tropical forest soils, e.g., soils from an old-growth lowland forest in Eastern Amazonia, Caxiuanã, Brazil (5.0–13.9 mg N kg^−1^ soil day^−1^)^17^ and in Indonesia (>5.0 mg N kg^−1^ soil day^−1^)^18^. However, as shown in Fig. 3, the average total gross rate of N mineralization (M_Nlab_ + M_Nrec_) in southern soils (3.04 mg N kg^−1^ d^−1^) was higher than that in northern soils (1.80 mg N kg^−1^ d^−1^; p < 0.05). The turnover rate of organic N was also significantly higher in southern soils than in northern soils (p < 0.05; Fig. 1). The N transformation rates obtained from laboratory-incubated soils were all measured at the same temperature. Taking into account the large difference in annual temperatures between the two regions (Table 1), we might expect that the N cycling rate *in situ* would be much faster in southern soils than in northern soils, although the N mineralization rate has been reported to be relatively insensitive to temperature^19^,^20^. Therefore, southern soils may provide greater opportunity for plants to take up N, even though the net mineralization rates and inorganic N contents are very similar in these two forest soils (Fig. 2 and Table 1).

The soil organic C and N concentrations and the ratios of organic C to N in the soil are thought to be the major factors that control soil N mineralization^21^,^22^,^23^. However, no significant relationship between gross mineralization rates and soil properties (e.g., soil organic C and N concentration, C to N ratio, and pH) was observed. The vegetation type (e.g., coniferous and broadleaf vegetation) may influence the N mineralization dynamics^10^ because the quantity and quality of soil organic matter are related to plant type and litter quality^17^. The mechanisms underlying the relatively rapid gross rates of organic N mineralization in southern soils vs northern soils need to be further explored.

For soils with high organic N mineralization and turnover rates to become N enriched, they have to develop a strong capacity for retaining inorganic N in soils. The majority of forest soils examined in this study were acidic, and all of the southern soils had pH ≤ 5.0 (Table 1). With such low soil pH, ammonia volatilization is almost completely suppressed, while NO_3_^−^ is easily lost via leaching and runoff, especially with high levels of precipitation, such as the region's average annual precipitation rate of ≥ 1,650 mm (Table 1). In our samples, the ammonium oxidation rates for all of the southern soils (0.14 ± 0.17 mg N kg^−1^ d^−1^) were much lower than those of northern soils (1.07 ± 1.57 mg N kg^−1^ d^−1^), while the nitrification capacities were 6% and 68%, respectively, in these two types of soils (Table 2 and Fig. 3). As a result, ammonium was much more dominant in southern soils than in northern soils. Therefore, ammonium being the dominant inorganic form of N is one mechanism by which southern soils retain inorganic N.

Both the ammonium oxidation rate and the nitrification capacity were exponentially correlated with soil pH (p < 0.01 and p < 0.01, respectively). These relationships highlight the importance of soil pH to the nitrification process^6^,^10^,^24^,^25^. Compared to northern soils, the lower ammonium oxidation rates and smaller nitrification capacities of southern soils could be attributed to the relatively low pH of these soils.

Moreover, our results show that NO_3_^−^ immobilization is a widespread process in southern acidic forest soils but that in the majority of northern soils, the rates of NO_3_^−^ immobilization and NO_3_^−^ capacity were low (Table 2). On average, the rate of immobilization of NO_3_^−^ into organic N accounted for 93% of the total nitrification rate (i.e., the ammonium oxidation rate plus the heterotrophic nitrification of organic N) in southern soils. In contrast, NO_3_^−^ immobilization accounted for only 3% of the total nitrification rate in northern soils (Fig. 3). DNRA occurred in all of the forest soils, including northern soils. Compared to NO_3_^−^ immobilization, however, the DNRA process was much less important for the retention of NO_3_^−^ in southern soils (accounting for 8% of the total NO_3_^−^ production rate), while the process was more important than NO_3_^−^ immobilization for the retention of NO_3_^−^ in northern soils (accounting for 22% of the total NO_3_^−^ production rate). The differences in NO_3_^−^ immobilization may further explain why the NO_3_^−^ concentrations in southern soils were much lower than those in northern soils (Table 1). In contrast to their high nitrification capacity, the denitrification potential was much lower in southern soils^13^. These results suggest that the competition between the denitrification and immobilization of NO_3_^−^ was also weaker in southern soils than in northern soils.

A number of previous studies have suggested that microorganisms generally prefer NH_4_^+^ as a N source^26^,^27^ and that NH_4_^+^ can inhibit the immobilization of nitrate, even at low concentrations^27^,^28^,^29^. This phenomenon appears to be true for temperate forest soils located in northern China but not for the humid subtropical soils of southern China. Our results show that NO_3_^−^ immobilization occurs universally and that the gross rate at which this process occurs is in many instances even higher than the total NH_4_^+^ production rate in southern soils. Previous studies have shown that microbial immobilization of NO_3_^−^ does occur^9^,^11^,^30^,^31^,^32^,^33^ and that fungi may preferentially utilize NO_3_^−^34,35. A previous investigation has also shown that the relative activity of fungi was enhanced in China's low latitude southern forest soils compared to northern forest soil^36^. Therefore, the observed NO_3_^−^ immobilization may be related to fungal activity. A comparison of the climates suggests that the capacity for NO_3_^−^ immobilization into organic N might be stronger in soils that have developed in warm, humid climate conditions because the soils formed under cold conditions had such a low capacity (e.g., the temperate zone in this study) (Table 2). In future studies, microbial factors governing these N enrichment processes should be elucidated.

## Methods

Five typical temperate forest sites located in northern China (defined as northern soil) and four typical subtropical forest sites located in southern China (defined as southern soil) were selected (Lat. 47°35′N to 19°04′N and Long. 109°31′E to 133°31′E, Table 3). All sites were located in nature reserves. Except for site 4, which was close to a city (within 100 km) and which had an N deposition rate of approximately 20 kg N ha^−1^ a^−1^^37^,^38^, all sites were far from urban or industrial areas, and the N deposition was low (<10 kg N ha^−1^ a^−1^)^39^,^40^,^41^. At each site, the typical forest ecosystem (i.e., the typical dominant vegetation) of the sample region was selected. The distance between different forest types at each site was more than 2000 m. Previous investigations have suggested that samples may be spatially correlated at 3.89 m to 18.5 m for 20 soil properties, but if they are farther apart, the soil samples are spatially independent^42^. The results of a hierarchical cluster analysis based on the main measured soil properties also showed that soil samples were independent in this study. From each forest type, soil samples were taken from three grids (approximately 4 m × 4 m) that were randomly placed in a representative 100 m × 100 m plot. From each grid, the O horizon, if present, was removed, and one subsample was then taken from the mineral A horizon (0–20 cm). Three subsamples were pooled together, sieved (2 mm), homogenized, and subsequently split into two subsamples. One subsample was stored at 4°C for the incubation studies, which were carried out within two months. The other subsample was air dried for the analysis of soil properties (Table 1). In total, 25 forest soils were sampled from northern and southern zones in East China. Eight soil samples were taken from northern China (samples 1–8), and 18 samples were taken from southern China (samples 9–20 and 21–25). Soil sampling was carried out in August 2009.

We employed a combination of ^15^N tracing experiments and full process-based N cycle models to quantify process-specific and pool-specific N transformation rates, which is the standard method for the quantification of N dynamics in soils^11^,^14^,^43^,^44^,^45^. For all samples, except 15–19, ^15^N tracing studies were carried out in the laboratory under controlled conditions. For samples 15–19, the ^15^N tracing experiments were conducted in the field.

For the ^15^N tracing laboratory experiments, we employed two NH_4_NO_3_ treatments (each with three repetitions). In the first, ammonium (^15^NH_4_NO_3_) was labeled with ^15^N at 20 atom% excess, and in the second, nitrate (NH_4_^15^NO_3_) was labeled. For each soil type, a series of 250-ml conical flasks was prepared, each containing 30 g of fresh soil. Two ml of ^15^NH_4_NO_3_ or NH_4_^15^NO_3_ solution was added to each conical flask at a rate of 7.14 μmol N g^−1^ soil (50 μg NH_4_^+^-N g^−1^ soil and 50 μg NO_3_^−^-N g^−1^ soil). The soil was adjusted to 60% water holding capacity (WHC) and incubated for 144 hours at 25°C. The conical flasks were sealed with silicone rubber stoppers. The samples were aerated by removing the stoppers for 1 hour every 2 days. The soils (three repetitions for each treatment) were extracted at 0.5, 24, 72, and 144 hours after fertilizer application to determine the concentrations and isotopic compositions of NH_4_^+^ and NO_3_^−^.

For the ^15^N tracing field experiments, we added fresh, sieved soil (100 g oven-dry) to cylinders with bottoms (4.7 cm diameter × 7.3 cm long) corresponding to the bulk density of each soil in the field. All soil cores were pre-incubated for 24 h at environmental temperatures before the addition of the ^15^N tracer. We then added either ^15^NH_4_NO_3_ or NH_4_^15^NO_3_ solution with ^15^N at 99.2 atom% excess. Each of the soil cores received N at a rate of 2 μg NH_4_^+^-N g^−1^ soil and 2 μg NO_3_^−^-N g^−1^ soil, consistent with the experimental conditions employed in previous studies in temperate and tropical forest ecosystems^12^.

The labeling solution was injected into the packed soil cores using the 5-needle injection technique^46^. Each core received 1.5 mL of the ^15^N solution, delivered in 15 injections of 0.1 mL each, to the top, middle, and bottom of the core. For each injection, a 5.6-cm, 18-gauge side-port needle with an attached syringe was inserted to a depth of approximately 5 cm into the soil, and the solution was gently released as the needle was slowly withdrawn. Using multiple injections in each core is the most appropriate way to ensure the uniform labeling of the inorganic N pools^47^. After labeling, all cores were returned to their original collection site and incubated in the field for 139 h. The mean daily temperature ranged from 20–25°C, and the soil water content was 50–55% WHC during the incubation period. Triplicate samples were randomly collected from each labeling treatment at 0.5, 40, 88, and 139 h after ^15^N labeling to determine the concentrations and isotopic compositions of NH_4_^+^ and NO_3_^−^.

Soil properties were measured by following the Soil Agro-Chemical Analysis procedures of Lu (2000)^48^. Soil pH was measured in a 1:5 (v/v) soil-to-water ratio using a DMP-2 mV/pH detector (Quark Ltd., Nanjing, China). Soil organic carbon (SOC) was analyzed by wet-digestion with H_2_SO_4_-K_2_Cr_2_O_7_, and total N was determined by semi-micro Kjeldahl digestion using Se, CuSO_4_, and K_2_SO_4_ as catalysts. Ammonium and NO_3_^−^ were extracted with 2 M KCl at a soil/solution ratio of 1:5 on a mechanical shaker for 60 minutes at 300 rpm and 25°C. The extracts were filtered through filter paper (Qualitative Filter Paper, BH92410262), and the concentrations of NH_4_^+^ and NO_3_^−^ were determined with a continuous-flow analyzer (Skalar, Breda, Netherlands).

For isotopic analysis, NH_4_^+^ and NO_3_^−^ were separated by distillation with magnesium oxide and Devarda's alloy^10^,^49^ (see **Supplementary information**). The isotopic compositions of NH_4_^+^ and NO_3_^−^ were determined using an automated C/N analyzer coupled to an isotope ratio mass spectrometer (Europa Scientific Integra, UK).

**Figure 4:**
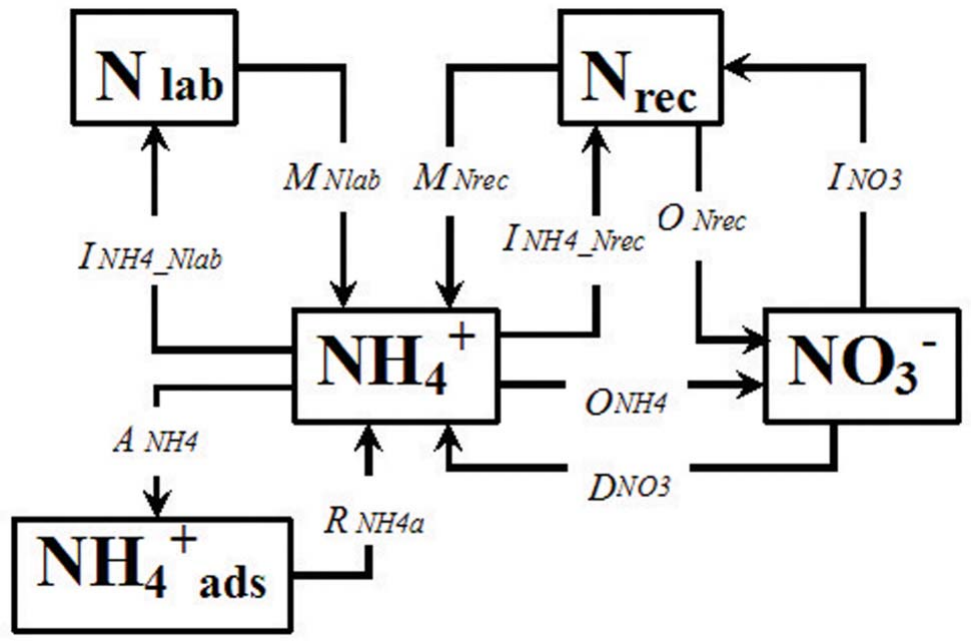
^15^N tracing model used for data analysis. *N_lab_* = labile soil organic N; *N_rec_* = recalcitrant soil organic N; NH_4_^+^ = ammonium; NO_3_^−^ = nitrate; NH_4_^+^_ads_ = adsorbed NH_4_^+^; *M_Nrec_*, mineralization of recalcitrant organic-N to NH_4_^+^; *M_Nlab_*, mineralization of labile organic-N to NH_4_^+^; *I_NH4_Nlab_*, immobilization of NH_4_^+^ to labile organic-N; *I_NH4_Nrec_*, immobilization of NH_4_^+^ to recalcitrant organic-N; *R_NH4ads_*, release of adsorbed NH_4_^+^; *A_NH4_*, adsorption of NH_4_^+^ on cation exchange sites; *O_NH4_*, oxidation of NH_4_^+^ to NO_3_^−^(autotrophic nitrification); *O_Nrec_*, oxidation of recalcitrant organic-N to NO_3_^−^ (heterotrophic nitrification); *I_NO3_*, immobilization of NO_3_^−^ to recalcitrant organic-N; and *D_NO3_*, dissimilatory NO_3_^−^ reduction to NH_4_^+^.

Simultaneous gross N transformations in soil were quantified using a process-based ^15^N tracing model (Fig. 4)^14^. The model analyzed 10 simultaneous gross N transformations. The transformation rates were calculated by zero-, first-order or Michaelis-Menten kinetics. Data supplied to the model included the concentrations and ^15^N excess values (averages ± standard deviations) of NH_4_^+^ and NO_3_^−^ from the two ^15^N treatments. The model calculated gross N transformation rates by simultaneously optimizing the kinetic parameters for the various N transformations by minimizing the misfit between modeled and observed NH_4_^+^ and NO_3_^−^ concentrations and their respective ^15^N enrichments. To identify the most appropriate model that best described the measured N dynamics, we used the procedure described by Rütting *et al*. (2008)^45^; the numbers of possible N transformations, kinetic settings and possible N pools were varied to find the best model. The final model (Fig. 4) was selected according to Akaike's Information Criterion (AIC), which takes into account the fit between observed and modeled data and the number of parameters^50^. Parameter optimization was carried out using the Metropolis algorithm (MCMC-MA; for further details on the algorithm see Müller *et al*. [2007]^14^). The misfit function between the observed and modeled data, *f*(**m**) (see eqt. 3 in Müller et al. 2007)^14^, takes into account the variance of the individual observations. Analyses using this parameter optimization concept in previous studies have shown that the mineralization of two conceptual organic-N pools produced realistic NH_4_^+^ dynamics^9^. The MCMC-MA routine was programmed in MatLab (Version 7.2, MathWorks Inc.), which calls models that are separately set up in Simulink (Version 6.4, MathWorks Inc.). Initial concentrations of the mineral N pools (^14^N and ^15^N pool sizes) were determined according to Müller *et al*. (2004)^51^. Concentrations of NH_4_^+^ and NO_3_^−^ were estimated for time zero by back-extrapolation of data at t = 0.5 h and t = 24 h (40 h for the in-field experiment). The difference between the applied NH_4_^+^ and the measured NH_4_^+^ was considered to be NH_4_^+^ that had been adsorbed shortly after N application to the NH_4_^+^ exchange sites (NH_4ads_). The optimization procedure resulted in a probability density function (PDF) for each parameter, from which the parameter averages and standard deviations were calculated^14^. Each analysis was carried out with three parallel sequences to identify adequate iteration numbers. Based on the kinetic settings and the final parameters, the average N transformation rates were calculated over the entire period and expressed in units of mg N kg^−1^ soil day^−1^ (Table 2).

Based on available studies of soil N cycling, this study focused on the total gross rates of mineralization (*M_Nrec_* + *M_Nlab_*), total nitrification (*O_NH4_* + *O_Nrec_*), autotrophic nitrification (*O_NH4_*), NO_3_^−^ (*I_NO3_*) and NH_4_^+^ (*I_NH4_Nlab_* + *I_NH4_Nrec_*) immobilization, and DNRA (*D_NO3_*). These parameters can elucidate the mechanisms operating in natural N-saturated and N-limited forests.

The ratio of the autotrophic nitrification rate (*O_NH4_*) to the mineralization rate (*M* = *M_Nrec_* + *M_Nlab_*) was defined as nitrification capacity (NC), indicating the soil's ability to transform available NH_4_^+^ to NO_3_^−^.

The ratio of total NO_3_^−^ consumption rate (NO_3_^−^ immobilization (I_NO3_) + DNRA) to total nitrification rate (N = O_NH4_ + O_Nrec_) was defined as the NO_3_^−^ retention capacity of the soil (NR).

The turnover rate of organic N = the total N/the gross N mineralization.

The net NO_3_^−^ production rate = the total nitrification rate – the total NO_3_^−^ consumption rate.

A hierarchical Cluster Analysis was used to distinguish the relatively homogeneous clusters of samples based on their primary soil properties. We used a Pearson correlation coefficient analysis to explore the relationship between measured variables. Calculations were made in SPSS 17.0 software for Windows. We compared soil characteristics and the N transformations between northern and southern soils using box-whisker plots and T-tests at a significance level of *p* < 0.05 in SPSS 17.0 software. We also analyzed the data using average measurements for each site to reduce the degree of pseudoreplication when we compared the soil N transformations between northern and southern soils and explored the relationships between variables. The results of the T-tests and Pearson correlation coefficient analysis using averaged data were consistent with the analysis treating each soil sample as a separate data point, except for the NC index (a T-test comparing NC between the northern and southern regions), which suggested that the comparison of N cycling between the northern and southern regions of China were reliable (see **Supplementary information**).

## Author Contributions

J.Z. and T.Z. participated in field sampling expeditions; T.Z. and W.Y. prepared the experimental set-up and scientific protocols. J.Z. wrote the manuscript and carried out data analysis; Z.C. and C.M. supervised the project. All authors reviewed the manuscript.

## Supplementary Material

Supplementary Information Supplementary_information

## Figures and Tables

**Table 1:** Sampling site characteristics and soil properties

Region	No.1)	pH	SOC2) g kg^−1^	TN g kg^−1^	C/N	NH_4_^+^ mg kg^−1^	NO_3_^−^ mg kg^−1^
Northern	1	5.0	30.1	2.3	13.3	20.5	10.6
	2	4.9	46.0	3.7	12.6	20.7	13.7
	3	4.8	48.6	3.7	13.2	3.1	10.2
	4	5.3	59.8	4.2	14.1	31.9	13.3
	5	4.9	83.0	8.4	9.9	9.9	21.8
	6	5.5	21.4	1.7	12.9	0.5	5.4
	7	7.4	46.7	4.0	11.7	6.3	68.5
	8	6.7	68.2	5.5	12.4	4.7	50.8
Southern	9	4.2	24.2	1.6	15.1	19.2	2.9
	10	4.3	24.1	1.1	21.9	18.8	3
	11	4.3	23.2	1.1	21.1	17.5	2.5
	12	4.2	62.8	3.1	20.3	45.1	1
	13	4.5	13.2	0.9	14.7	93.2	10
	14	4.6	30.4	2.3	13.3	16.2	3.9
	15	4.6	30.4	2.3	13.3	16.3	3.9
	16	4.1	68.2	3.9	17.7	33.4	4.2
	17	4.3	29.3	2.7	10.7	8.6	7.7
	18	4.3	33.3	3.0	11.1	16.1	6.1
	19	4.7	29.1	1.8	16.5	26.3	7.1
	20	4.6	18	0.6	29.2	2.1	1.7
	21	4.9	16	0.7	24.1	2.2	2
	22	4.7	18.1	0.8	21.6	2.4	2.1
	23	4.3	53.5	1.4	39.6	3.8	1.4
	24	4.5	56.2	2.4	23.8	23.8	2.5
	25	4.6	43.1	3	14.5	24.4	5.4
Northern soils3)		5.6b	50.5a	4.2b	12.5a	12.2a	24.3a
Southern soils (lab incubation)		4.5a	31.9a	1.58a	21.6b	22.4a	3.2a
Southern soils (field incubation)4)		4.4a	38.1a	2.7ab	13.9a	20.1a	5.8a

^1)^No., site number according to soil site listed from north to south.

^2)^SOC, soil organic C; TN, total N.

^3)^Identical letters indicate no significant differences in the average values.

^4)^Gross N transformation rates in soils 15–19 were determined by field incubation.

**Table 2:** Gross N transformation rates in forest soils (0–20 cm) estimated using a ^15^N tracing model

Region	No1)	M2)	I_NH4_	TNi	O_NH4_	I_NO3_	DNRA	NC	NR
Northern	1	2.16 ± 0.15	0.31 ± 0.14	0.22 ± 0.02	0.22 ± 0.04	0	0.11 ± 0.03	0.10 ± 0.05	0.50 ± 0.09
	2	2.14 ± 0.09	0.26 ± 0.08	0.07 ± 0.01	0.07 ± 0.01	0	0.04 ± 0.02	0.03 ± 0.01	0.57 ± 0.18
	3	1.02 ± 0.06	0	0.14 ± 0.00	0.13 ± 0.00	0	0.08 ± 0.01	0.13 ± 0.00	0.57 ± 0.04
	4	2.29 ± 0.17	0	0.88 ± 0.05	0.19 ± 0.02	0	0.02 ± 0.01	0.08 ± 0.01	0.02 ± 0.01
	5	2.21 ± 0.12	1.53 ± 0.53	0.85 ± 0.05	0.39 ± 0.00	0	0.01 ± 0.00	0.18 ± 0.01	0.01 ± 0.00
	6	1.62 ± 0.11	0.01 ± 0.01	1.41 ± 0.16	0.80 ± 0.03	0.31 ± 0.27	0.03 ± 0.02	0.50 ± 0.02	0.24 ± 0.11
	7	1.87 ± 0.23	2.58 ± 0.38	4.53 ± 0.03	4.53 ± 0.05	0	0.07 ± 0.04	2.43 ± 0.16	0.02 ± 0.01
	8	1.12 ± 0.05	0.01 ± 0.00	2.46 ± 0.12	2.24 ± 0.07	0	0.01 ± 0.01	2.00 ± 0.15	0
Southern	9	3.95 ± 0.11	1.94 ± 0.06	0.49 ± 0.15	0.02 ± 0.01	0.55 ± 0.16	0.01 ± 0.01	0.004 ± 0.00	1.14 ± 0.40
	10	1.52 ± 0.12	0.22 ± 0.12	0.81 ± 0.04	0.05 ± 0.03	0.69 ± 0.05	0.02 ± 0.01	0.03 ± 0.02	0.88 ± 0.06
	11	1.93 ± 0.15	0.35 ± 0.22	0.91 ± 0.15	0.05 ± 0.01	0.76 ± 0.02	0.02 ± 0.01	0.03 ± 0.03	0.86 ± 0.14
	12	3.70 ± 0.12	0.94 ± 0.12	0.06 ± 0.00	0.05 ± 0.01	0.11 ± 0.03	0.02 ± 0.00	0.01 ± 0.01	2.17 ± 0.31
	13	4.63 ± 0.19	2.34 ± 0.50	0.49 ± 0.02	0.23 ± 0.02	0.30 ± 0.03	0.04 ± 0.00	0.05 ± 0.01	0.69 ± 0.05
	14	2.72 ± 0.08	2.25 ± 0.53	0.93 ± 0.07	0.50 ± 0.01	0.70 ± 0.03	0.05 ± 0.01	0.18 ± 0.04	0.81 ± 0.06
	15	3.52 ± 0.18	2.25 ± 0.19	0.93 ± 0.02	0.51 ± 0.03	0.69 ± 0.03	0.05 ± 0.00	0.14 ± 0.02	0.80 ± 0.03
	16	2.29 ± 0.16	0.06 ± 0.02	1.00 ± 0.04	0.19 ± 0.04	0.74 ± 0.04	0.17 ± 0.01	0.09 ± 0.06	0.91 ± 0.04
	17	9.20 ± 0.43	4.99 ± 1.40	0.66 ± 0.05	0.65 ± 0.08	0.11 ± 0.06	0.35 ± 0.04	0.07 ± 0.02	0.70 ± 0.08
	18	5.79 ± 0.65	6.17 ± 1.05	0.52 ± 0.04	0.37 ± 0.02	0.25 ± 0.12	0.29 ± 0.02	0.06 ± 0.00	1.04 ± 0.15
	19	3.37 ± 0.35	0	1.14 ± 0.08	0.12 ± 0.02	0.59 ± 0.16	0.05 ± 0.01	0.04 ± 0.01	0.56 ± 0.09
	20	2.41 ± 0.36	2.33 ± 0.49	0.90 ± 0.06	0.13 ± 0.01	0.65 ± 0.13	0.06 ± 0.01	0.05 ± 0.01	1.63 ± 0.16
	21	3.54 ± 0.22	3.74 ± 0.47	0.72 ± 0.04	0	0.22 ± 0.01	0.07 ± 0.01	0	1.56 ± 0.02
	22	3.46 ± 0.21	2.88 ± 0.26	1.97 ± 0.04	0.04 ± 0.00	1.71 ± 0.09	0.07 ± 0.01	0.01 ± 0.00	0.90 ± 0.05
	23	3.01 ± 0.37	2.69 ± 0.27	0.76 ± 0.10	0.07 ± 0.01	0.91 ± 0.18	0.06 ± 0.01	0.02 ± 0.00	1.27 ± 0.29
	24	2.25 ± 0.16	0.30 ± 0.13	0.81 ± 0.10	0.12 ± 0.01	0.42 ± 0.18	0.04 ± 0.01	0.05 ± 0.01	1.93 ± 0.23
	25	3.33 ± 0.17	1.62 ± 0.38	0.52 ± 0.03	0.47 ± 0.02	0.75 ± 0.07	0	0.14 ± 0.01	1.44 ± 0.11
Northern soils3)		1.80a	0.59a	1.32a	1.07b	0.04a	0.05a	0.68b	0.24a
Southern soils (lab incubation)		3.04b	1.80a	0.78a	0.14a	0.65b	0.04a	0.05a	1.27b
Southern soils (field incubation)4)		4.83b	2.69ab	0.85a	0.37a	0.48b	0.18b	0.08a	0.80b

^1)^No., site number according to soil site listed from north to south.

^2)^M, mineralization rate of organic N pool; I_NH4_, total immobilization of NH_4_^+^; TNi, total nitrification rate (autotrophic nitrification + heterotrophic nitrification); O_NH4_, autotrophic nitrification; I_NO3_, immobilization of NO_3_^−^; DNRA, dissimilatory NO_3_^−^ reduction to NH_4_^+^; NC, nitrification capacity; NR, NO_3_^−^ retention capacity.

3)Identical letters indicate no significant differences in the average values between groups.

4)Gross N transformation rates in soils 15–19 were determined by field incubation.

**Table 3:** The dominant vegetation, soil type and land use history characteristics in the study sites

Region	Site	Sample1)	Lat. (N)	Lon. (E)	Tem.2) °C	Pre. mm	Dominant vegetation	Soil type	Land use history
Northern	1	1	47°35′	133°31′	1.9	600	*Betula platyphylla*	Cryumbreps	Natural
	2	2	47°07′	128°50′	1.0	723	*Betula platyphylla*	Cryumbreps	Natural
		3	47°07′	128°50′	1.0	723	*Pinus koraiensis*	Cryumbreps	Natural
	3	4	42°24′	128°28′	3.0	695	*Betula platyphylla*	Cryumbreps	Natural
		5	42°24′	128°28′	3.0	695	*Pinus koraiensis*	Cryumbreps	Natural
	4	6	41°48′	123°24′	7.4	711	*Quercus liaotungensis*	Hapludults	Plantation approximately 50 years
	5	7	36°35′	117°51′	11.9	694	*Ulmus pumila*	Hapludults	Plantation approximately 100 years
		8	36°35′	117°51′	11.9	694	*Platycladus orientalis*	Hapludults	Plantation approximately 100 years
Southern	6	9	27°59′	117°25′	17.6	1788	*Pinus massoniana*	Haplustalfs	Natural
		10	27°59′	117°25′	17.6	1788	*Pinus massoniana*	Haplustalfs	Natural
		11	27°59′	117°25′	17.6	1788	*Pinus massoniana*	Haplustalfs	Natural
		12	27°59′	117°25′	17.6	1788	*Cinnamomum camphora*	Haplustalfs	Natural
		13	27°59′	117°25′	17.6	1788	*Pinus massoniana*	Haplustalfs	Natural
	7	14	27°03′	118°09′	19.4	1700	*Castanobsis sclerophylla*	Haplustalfs	Natural
		15	27°03′	118°09′	19.4	1700	*Castanobsis sclerophylla*	Haplustalfs	Natural
		16	27°03′	118°09′	19.4	1700	*Altingia gralilipes*	Haplustalfs	Natural
		17	27°03′	118°09′	19.4	1700	*Cinnamomum chekiangense*	Haplustalfs	Natural
		18	27°03′	118°09′	19.4	1700	*Tsoongiodendron odorum*	Haplustalfs	Natural
		19	27°03′	118°09′	19.4	1700	*Castanopsis fargesii*	Haplustalfs	Natural
	8	20	25°24′	101°28′	14.7	1300	*Pinus yunnanensis faranch*	Haplustalfs	Plantation approximately 30 years
		21	25°24′	101°28′	14.7	1300	*Pinus yunnanensis faranch and Cyclobalanopsis lobbii*	Haplustalfs	Plantation approximately 40 years
		22	25°24′	101°28′	14.7	1300	*Cyclobalanopsis lobbii*	Haplustalfs	Plantation approximately 50 years
		23	25°24′	101°28′	14.7	1300	*Pinus yunnanensis faranch and Cyclobalanopsis lobbii*	Haplustalfs	Natural
		24	25°24′	101°28′	14.7	1300	*Pinus yunnanensis faranch and Cyclobalanopsis lobbii*	Haplustalfs	Natural
	9	25	23°42′	114°11′	21	2133	*Castanobsis sclerophylla*	Paleudalfs	Natural

^1)^Sample, number according to soil site listed from north to south.

^2)^Tem, mean annual temperature; Pre, annual precipitation.
