# Supplementary material for: Mechanisms for the retention of inorganic N in acidic forest soils of southern China
Source: Sci Rep. 2013 Aug 2;3:2342. doi: 10.1038/srep02342 (PMC3731645; doi:10.1038/srep02342)
Supplement: Supplementary Information — Supplementary_information [file srep02342-s1.pdf]

Title: Mechanisms for retention of inorganic N in acid forest soils in southern China

Jin-bo Zhang[1,2], Zu-cong Cai[1,2*], Tong-bin Zhu[1,2], Wen-yan Yang[1], Christoph Müller[3]

[1]School of Geography Sciences, Nanjing Normal University, Nanjing 210023, China

[2]State Key Laboratory of Soil and Sustainable Agriculture, Institute of Soil Science, Chinese Academy of Sciences

[3] Department of Plant Ecology, Justus-Liebig University Giessen, Heinrich-Buff-Ring 26, 35392 Giessen, Germany

[*]E-mail: zccai@njnu.edu.cn (Cai);

Separation of NH_4^+ and NO_3^-

For isotopic analysis, NH_4^+ and NO_3^- were separated by distillation with magnesium oxide and Devarda's alloy[1,2]. In detail, a portion of the extract was steam-distilled with MgO in a steam distillation system to isolate the NH_4^+. The sample in the flask was then distilled again after the addition of Devarda's alloy to isolate the NO_3^-. The liberated NH_3 was trapped using a boric acid solution. The trapped N was acidified and converted to $(NH_4)_2SO_4$ using a 0.02 mol L^{-1} H_2SO_4 solution. The H_2SO_4 solution containing NH_4^+ was then evaporated to dryness at 65°C in an oven and analysed for ^{15}N abundance. Before separating NH_4^+ and NO_3^- in the extract using a steam distillation system, the recovery of NH_4^+ and NO_3^- from a standard solution (1 g NH_4^+-N L^{-1} and 1 g NO_3^--N L^{-1}) was determined. The results showed that almost all of the NH_4^+-N in the solution could be recovered (>99%), and the recovery of NO_3^- was >95%.

Comparison of dilution and ^{15}N tracing model methods

To verify the utility of the ^{15}N tracing model for simulating the gross rates of N transformation that occur simultaneously, we compared the results obtained using the ^{15}N dilution method[3] with those obtained using the ^{15}N tracing method employed in this study.

The gross rates of mineralization, nitrate production, and NO_3^- consumption per time interval and the average of all time intervals were calculated using the method of Kirkam and Bathalomew (1954)[3]. The results showed the calculated (dilution technique) and modelled (^{15}N tracing technique) mineralization and NO_3^- production

rates were similar ($p < 0.01$; Fig. 2A, B). Based on the generally strong agreement between the values, we were confident enough to use the modelled rates, which provided details on the N transformation mechanisms and therefore a much more detailed view of actual N dynamics. Therefore, the modelled rates were used to compare soil N transformations between temperate and subtropical-tropical forest soils in the present investigation.

Samples 14 and 15 were from the same core, but the ^{15}N tracing experiment was carried out in the laboratory for soil 14 (added 50 μg N g^{-1} soil) and in the field for soil 15 (added 2 μg N g^{-1} soil). The results showed that the N transformation rates determined in the laboratory and in the field were comparable (Table 2 in article), and thus the N transformations were not stimulated in the laboratory due to higher N application in the studied acid forest soils. The laboratory studies can provide essential information to mechanistically understand the observed N cycling process in the field[4,5], despite some reported problems[6]. Nitrogen enrichment in humid subtropical soils could therefore be explained using data obtained by comparing the gross rates of N transformation in laboratory-incubated soils with those of temperate soils (Fig. 3 in article).

Previous studies, where the ^{15}N tracing approach (section 2.4 in article) was used, showed that N consumption/production rates can be separated into process-specific gross rates to provide more details about soil N transformations, despite the fact that there is still some uncertainty about the general nature of the application of the ^{15}N tracing model[5,7,8,9,10,11]. It should be stressed that, in general, individual gross N

transformation rates cannot be directly measured but can only be quantified with the help of analytic models, the most basic of which was presented by Kirkham and Bartholomew (1954)[7]. Subsequently, more complete and realistic analytic models have been developed. The latest are numerical ^{15}N tracing models in which the N transformation rate parameters are identified by non-linear optimization techniques[7,10]. We decided to calculate the total NH_4^+ and NO_3^- production and consumption rates via the dilution method (Kirkam and Bathalomew 1954)[7] and the current ^{15}N tracing model. Comparison of the model results showed that the two methods provided comparable results for mineralization and NO_3^- production rates ($p < 0.01$; Fig. 1A, B). The advantage of the ^{15}N tracing approach is that not only pool-specific gross N rates, but also individual N rates, can be determined. Furthermore, ^{15}N tracing models overcome the restrictions of zero-order kinetic rates that are assumed in the dilution approach. First-order and Michaelis-Menten kinetics are more realistic because they take into account a non-linear behaviour with respect to changing N concentrations. Discrepancies between the two methods may be associated with the use of different kinetics[11] (see also the result for NO_3^- immobilization for the two neutral temperate forest soils [soil 7, 8], Fig. 1C).

The results of analysis using average of soil properties and gross N transformation rates at each site

Table 1 The result of T-test analysis using average of soil gross N transformation rates at each site comparing between northern and southern region

Transformation process*	Levene's Test for Equality of Variances			t-test for Equality of Means			95% Confidence Interval of the Difference	
		F	Sig.	t	Degrees of freedom	Sig.	Lower	Upper
M	Equal variances assumed	1.346	0.284	-4.682	7	0.002	-2.49	-0.82
	Equal variances not assumed			-4.345	4.256	0.011	-2.68	-0.62
TNi	Equal variances assumed	3.552	0.101	0.666	7	0.527	-1.16	2.07
	Equal variances not assumed			0.749	4.342	0.492	-1.18	2.09
O_{NH4}	Equal variances assumed	3.332	0.111	0.999	7	0.351	-0.97	2.38
	Equal variances not assumed			1.127	4.224	0.32	-1.00	2.41
I_{NO3}	Equal variances assumed	0.64	0.45	-5.818	7	0.001	-0.80	-0.34
	Equal variances not assumed			-5.735	6.17	0.001	-0.81	-0.33
NC	Equal variances assumed	4.32	0.076	1.145	7	0.29	-0.57	1.63
	Equal variances not assumed			1.297	4.038	0.264	-0.60	1.67

NR	Equal variances assumed	0.022	0.886	-5.002	7	0.002	-1.38	-0.49
	Equal variances not assumed			-4.923	6.118	0.003	-1.40	-0.47
Turnover time of organic N	Equal variances assumed	14.97	0.006	2.735	7	0.029	212.06	2921.27
	Equal variances not assumed			3.075	4.347	0.033	195.47	2937.87
DNRA	Equal variances assumed	1.195	0.31	-0.276	7	0.791	-0.10	0.08
	Equal variances not assumed			-0.256	4.287	0.81	-0.12	0.10

* M, mineralization rate of organic N pool; TNi, total nitrification rate (autotrophic nitrification + heterotrophic nitrification); O_{NH4}, autotrophic nitrification; I_{NO3}, immobilization of NO_3^-; NC, nitrification capacity; NR, NO_3^- retention capacity; DNRA, dissimilatory NO_3^- reduction to NH_4^+.

Table 2 Pearson correlation coefficient between soil pH and autotrophic nitrification (O_{NH4}) nitrification capacity (NC) by using site-averages

Factor		O_{NH4}	NC
pH	Pearson Correlation	0.934**	0.950**
	Sig. (2-tailed)	0.000	0.000
	N	9	9

**. Correlation is significant at the 0.01 level (2-tailed).

Table 3 Pearson correlation coefficient between gross rates of mineralization and NH_4^+ immobilization by using site-averages

Factor		NH_4^+ immobilization rates
Gross mineralization	Pearson Correlation	0.799**
	Sig. (2-tailed)	0.010
	N	9

**. Correlation is significant at the 0.01 level (2-tailed).

References

1. Feast, N.A. & Dennis, P.E. (1996). A comparison of methods for nitrogen isotope analysis of groundwater. *Chem. Geol.*, 129, 167-171.

2. Zhang, J.B., Zhu, T.B., Cai, Z.C. & Müller, C. (2011). Nitrogen cycling in forest soils across climate gradients in Eastern China. *Plant Soil*, 342, 419 - 432.

3. Kirkham, D. & Bartholomew, W.V. (1954). Equations for following nutrient transformations in soil, utilizing tracer data. *Soil Sci. Soc. Am. Pro.*, 18, 33–34.

4. Huygens, D., Boeckx, P., Templer, P., Paulino, L., Cleemput, O.V., Oyarzún, C., Müller, C. & Godoy, R. (2008). Mechanisms for retention of bioavailable nitrogen in volcanic rainforest soils. *Nat. Geosci.*, 1, 543-548.

5. Huygens, D., Rütting, T., Boeckx, P., Van Cleemput, O., Godoy, R. & Müller, C. (2007). Soil nitrogen conservation mechanisms in a pristine south Chilean *Nothofagus* forest ecosystem. *Soil Biol. Biochem.*, 39, 2448–2458.

6. Arnold, J., Corre, M.D., Veldkamp, E. (2008). Cold storage and laboratory incubation of intact soil cores do not reflect in-situ nitrogen cycling rates of tropical forest soils. *Soil Biol. Biochem.*, 40, 2480–2483.

7. Mary, B., Recous, S., Robin, D. (1998). A model for calculating nitrogen fluxes in soil using 15N tracing. *Soil Biol. Biochem.*, 30, 1963–1979.

8. Paterson, E. (2003). Importance of rhizodeposition in the coupling of plant and microbial productivity. *Eur. J. Soil Sci.*, 54,741–750.

9. Luxhøi, J., Jensen, L.S. (2005). Gross N mineralization–immobilization rates in heterogeneous intact soil cores can be estimated without marked error. *Biol. Fertil. Soils*, 41, 280–283.

10. Müller, C., Rütting, T., Kattge, J., Laughlin, R.J. & Stevens, R.J. (2007). Estimation of parameters in complex ^{15}N tracing models via Monte Carlo sampling. *Soil Biol. Biochem.*, 39, 715–726.

11. Rütting, T. & Müller, C. (2007). ^{15}N tracing models with a Monte Carlo optimization procedure provide new insights on gross N transformations in soils. *Soil Biol. Biochem.*, 39, 2351–2361

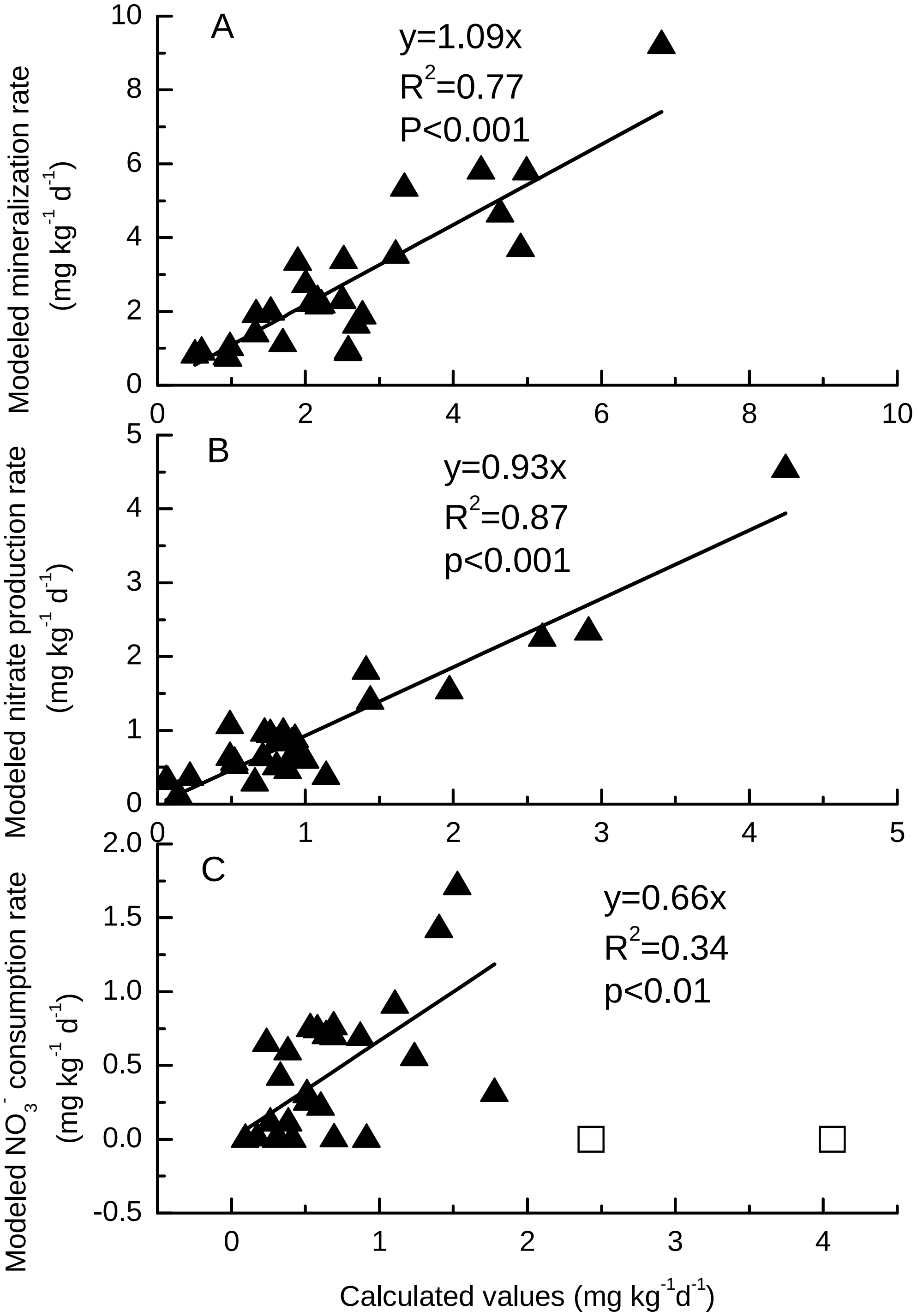

Fig. 1 Comparison of modelled and calculated total mineralization (A), nitrification (B) and NO_3^- immobilization rates (C; mg N kg^{-1} d^{-1})

The modelled rates were simulated using the ^{15}N tracing model (Müller et al., 2007)[10], and the calculated rates were obtained using the method of Kirkam and Bathalomew (1954)[7].
